# Supplementary material for: Naringenin and Quercetin Exert Contradictory Cytoprotective and Cytotoxic Effects on Tamoxifen-Induced Apoptosis in HepG2 Cells
Source: Nutrients. 2022 Dec 19;14(24):5394. doi: 10.3390/nu14245394 (PMC9783584; doi:10.3390/nu14245394)
Supplement: Supplementary file 1 [file nutrients-14-05394-s001.zip › nutrients-1913556-supplementary.pdf]

nutrients

Article

Naringenin and Quercetin Exert Contradictory Cytoprotective and Cytotoxic Effects on Tamoxifen-Induced Apoptosis in HepG2 Cells

Zhixiang Xu [1,2], Yue Jia [1], Jun Liu [1], Xiaomin Ren [1], Xiaoxia Yang [1,*], Xueshan Xia [2] and Xuejun Pan [1,*]

[1] Faculty of Environmental Science & Engineering, Kunming University of Science and Technology, Kunming 650500, China
[2] Faculty of Life Science & Technology, Kunming University of Science and Technology, Kunming 650500, China
* Correspondence: 20212007@kust.edu.cn (X.Y.); xjpan@kust.edu.cn (X.P.); Tel./Fax: +86-871-65920510 (X.Y.); Tel.: +86-871-65916001 (X.P.); Fax: +86-871-65916002 (X.P.)

Citation: Xu, Z.; Jia, Y.; Liu, J.; Ren, X.; Yang, X.; Xia, X.; Pan, X. Naringenin and Quercetin Exert Contradictory Cytoprotective and Cytotoxic Effects on Tamoxifen-Induced Apoptosis in HepG2 Cells. *Nutrients* **2022**, *14*, 5394. https://doi.org/10.3390/nu14245394

Academic Editor: Naoki Tanaka

Received: 29 August 2022
Accepted: 6 October 2022
Published: 19 December 2022

Publisher's Note: MDPI stays neutral with regard to jurisdictional claims in published maps and institutional affiliations.

Copyright: © 2022 by the authors. Licensee MDPI, Basel, Switzerland. This article is an open access article distributed under the terms and conditions of the Creative Commons Attribution (CC BY) license (https://creativecommons.org/licenses/by/4.0/).

Supplementary Materials

Figures

Figure S1: Tamoxifen, naringenin and quercetin changed the cellular morphology and adhering capacity

Figure S2: Representative bright-field photographs of wound closure for cell migration determination

Figure S3: Representative flow cytometry images with PI staining for cell cycle determination

Figure S4: Representative flow cytometry images with Annexin V-FITC/PI double staining for cell apoptosis determination

Figure S5: Representative flow cytometry images with PI staining for cell apoptosis determination

Figure S6: Representative flow cytometry images with JC-1 staining for $\Delta\Psi m$ determination

Tables

Table S1: Nucleotide sequences of primers used for RT-qPCR analysis

Table S2: IC50 (μM) of tamoxifen, naringenin and quercetin at different exposure times were determined using the CCK-8 assay

Table S1. Nucleotide sequences of primers used for RT-qPCR analysis.

Gene	Forward Primer	Reverse Primer
MMP-2	5′-GCCTCTCCTGACATTGACCTTGG-3′	5′-CACCACGGATCTGAGCGATGC-3′
MMP-9	5′-TCCTGGTGCTCCTGGTGCTG-3′	5′-CTGCCTGTCGGTGAGATTGGTTC-3′
N-cadherin	5′-TGCCATCATTGCCATCCTGCTC-3′	5′-AGTCCTGGTCTTCTTCTCCTCCAC-3′
E-cadherin	5′-GCTCTTCCAGGAACCTCTGTGATG-3′	5′-AAGCGATGGCGGCATTGTAGG-3′
cyclin D1	5′-CAGAAGTGCGAGGAGGAGGT-3′	5′-TAGAGGCCACGAACATGCAA-3
cyclin E	5′-AGCCACCTCCAGACACCAGT-3′	5′-AGAACACCTGCGAGGAGAGC-3′
p21	5′-TCCAGCGACCTTCCTCATCCAC-3′	5′-TCCATAGCCTCTACTGCCACCATC-3′
p53	5′-ACTAAGCGAGCACTGTCCAACAAC-3′	5′-GCCTCATTCAGCTCTCGGAACATC-3′
Bcl-2	5′-GCTTATCGTCGTGTGAA-3′	5′-GGGATGTGCTCGGTAAGTGT-3′
Bax	5′-GGGATCATGAGGTCAGGAGA-3′	5′-CCCGAGTAGCTGGGACTACA-3′
β-actin	5′-TGAGCGCGGCTACAGCTT-3′	5′-TCCTTAATGTCACGCACGATTT-3′

Table S2. IC50 (μM) of tamoxifen, naringenin and quercetin at different exposure times were determined using the CCK-8 assay.

Compounds	24 h	48 h	72 h
Tamoxifen	59.20 ± 1.90	48.67 ± 1.41	37.07 ± 0.93
Naringenin	537.90 ± 17.67	516.50 ± 14.94	403.80 ± 10.40
Quercetin	488.40 ± 6.50	323.50 ± 1.99	270.50 ± 1.34

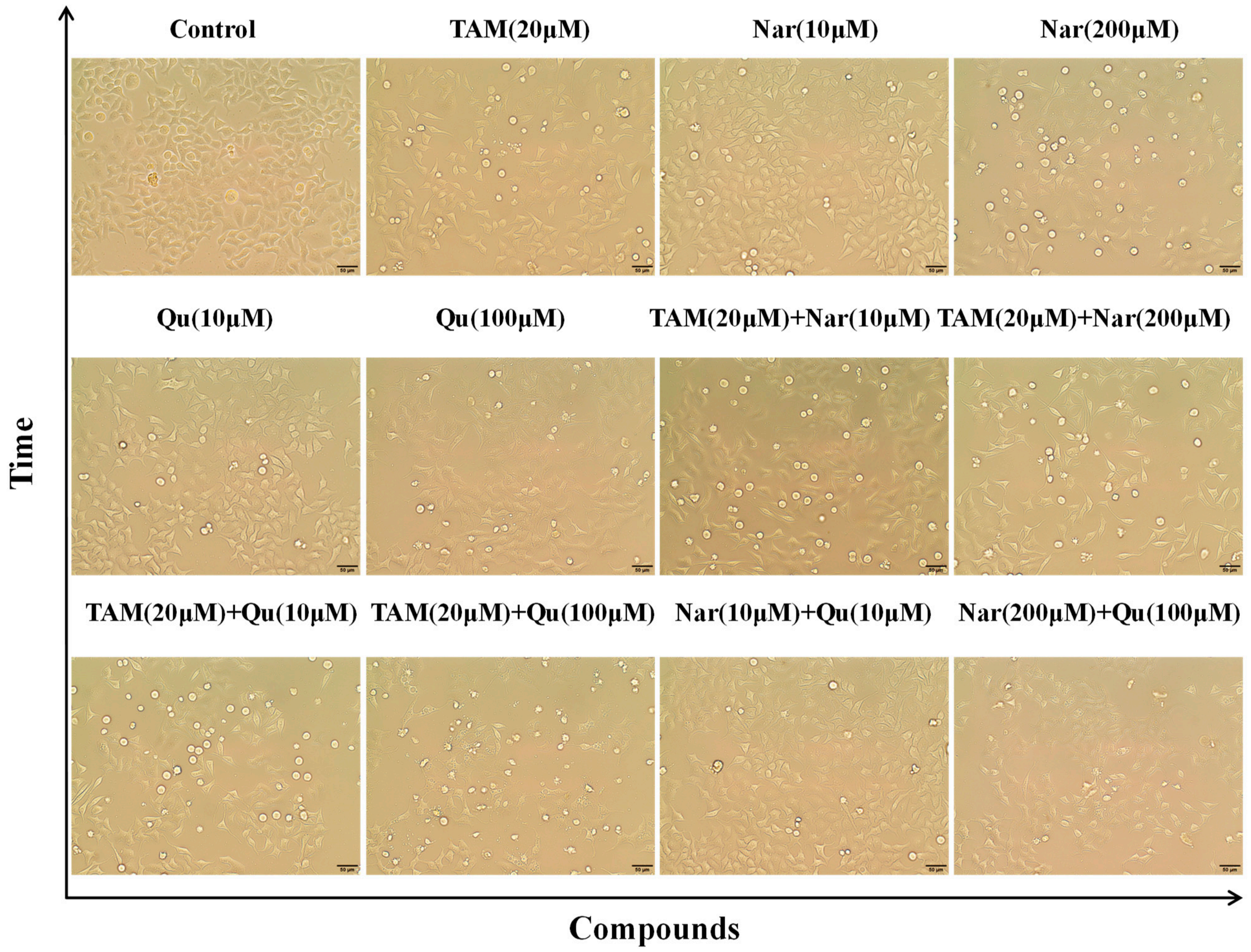

Figure S1. Tamoxifen, naringenin and quercetin changed the cellular morphology and adhering capacity. HepG2 cells were exposed to tamoxifen (20 μM), naringenin (10 and 200 μM) and quercetin (10 and 100 μM) either alone or in combination for 24 h, and the morphological changes were observed at 200× magnifications using an inverted fluorescence microscope.

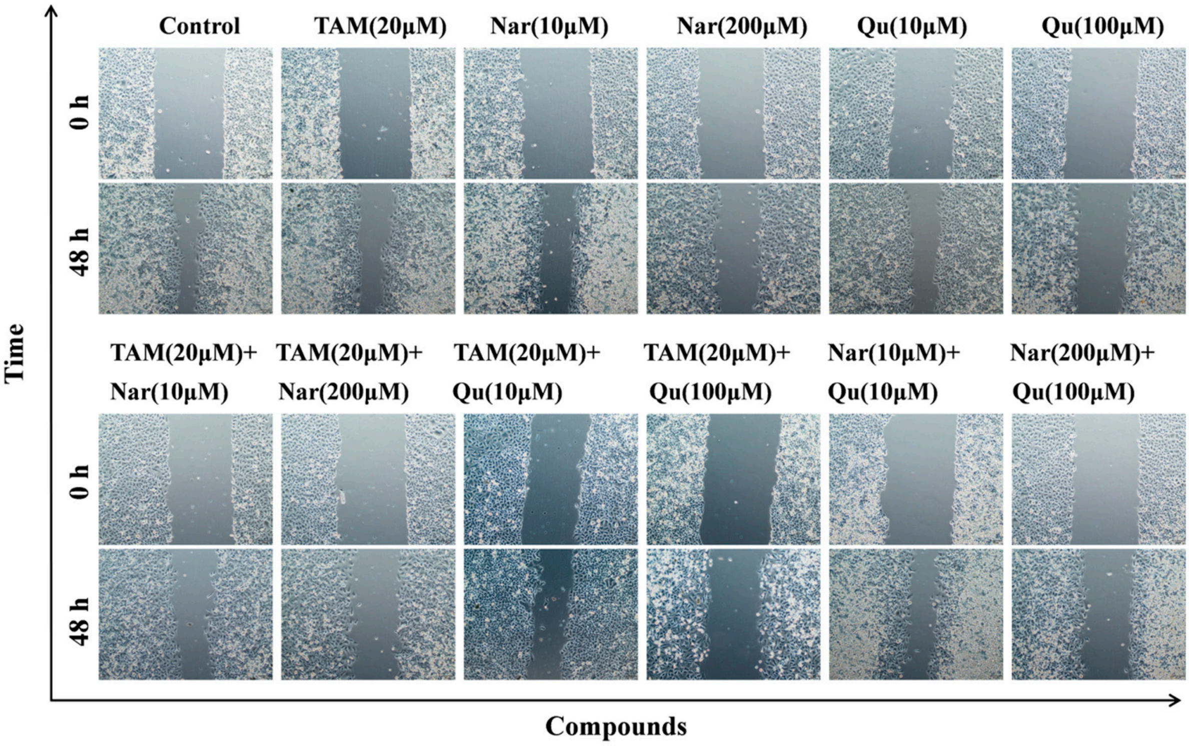

Figure S2. Representative bright-field photographs of wound closure for cell migration determination. HepG2 cells were exposed to tamoxifen (20 μM), naringenin (10 and 200 μM) and quercetin (10 and 100 μM) either alone or in combination for 48 h. The wound closure was photographed at 0 and 48 h using an inverted fluorescence microscope at a magnification of 100×.

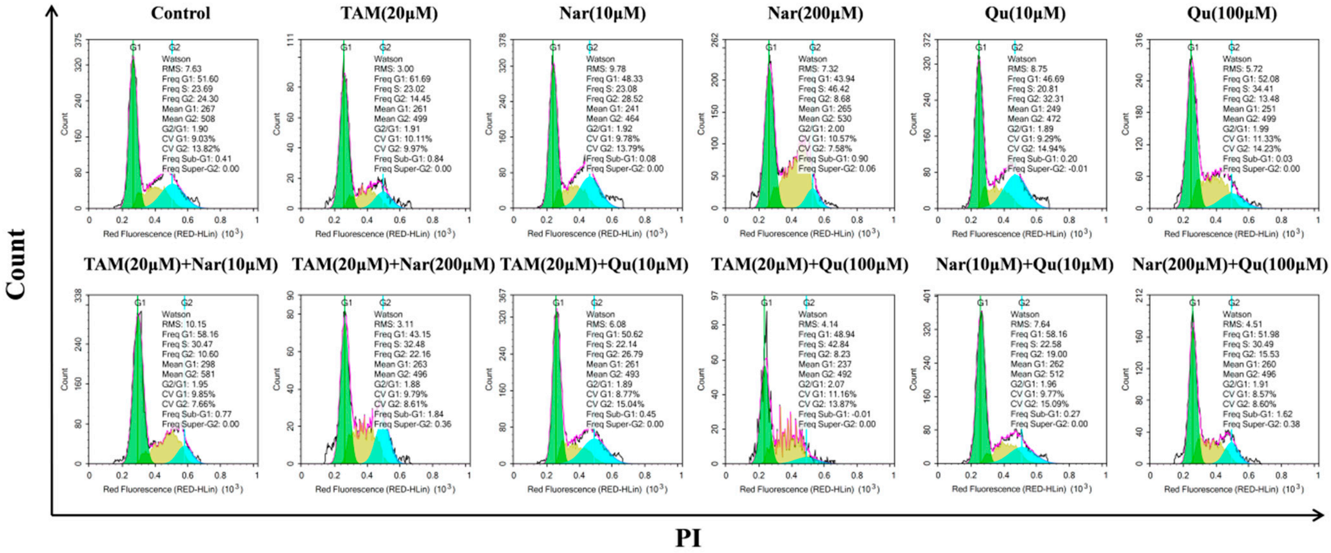

Figure S3. Representative flow cytometry images with PI staining for cell cycle determination. HepG2 cells were exposed to tamoxifen (20 μM), naringenin (10 and 200 μM) and quercetin (10 and 100 μM) either alone or in combination for 24 h. The cell cycle distribution (i.e., G0/G1, S and G2/M phases) was calculated with PI staining using flow cytometry.

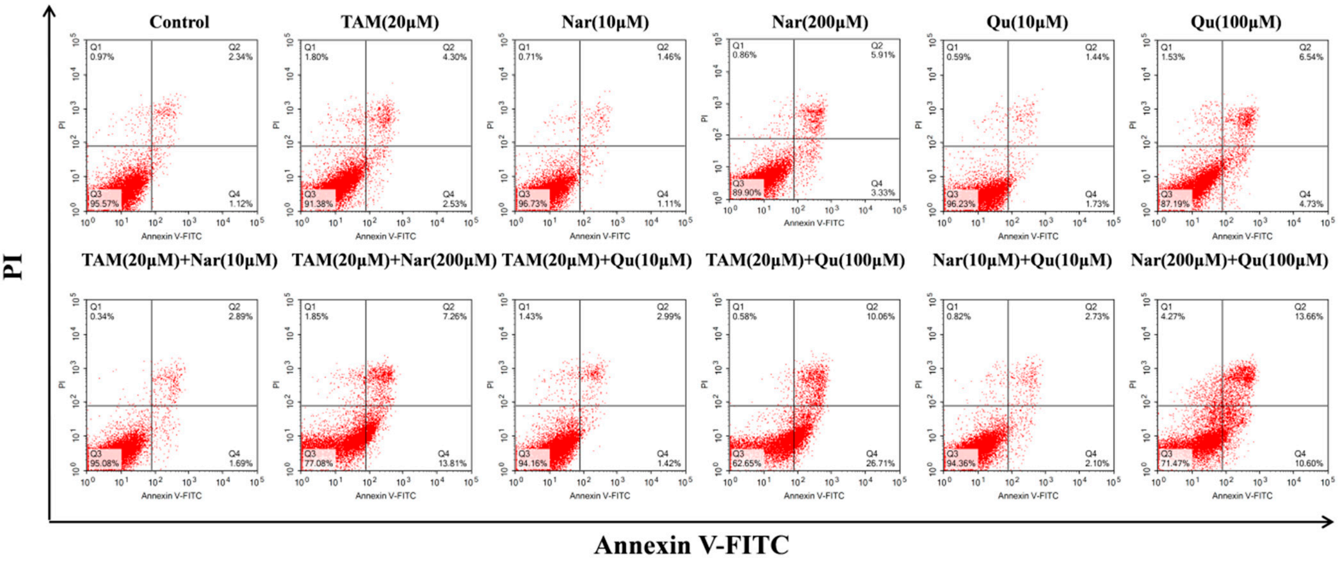

Figure S4. Representative flow cytometry images with Annexin V-FITC/PI double staining for cell apoptosis determination. HepG2 cells were exposed to tamoxifen (20 μM), naringenin (10 and 200 μM) and quercetin (10 and 100 μM) either alone or in combination for 24 h. The apoptosis proportion (i.e., early apoptosis, late apoptosis and total apoptosis) were then calculated with Annexin V-FITC/PI double staining using flow cytometry.

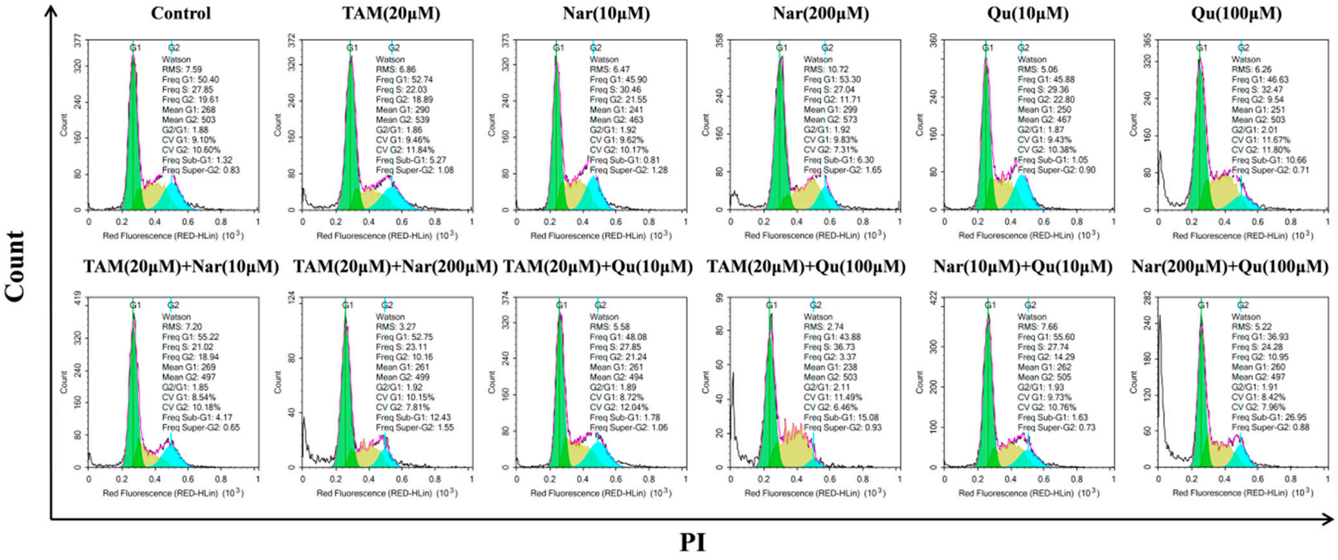

Figure S5. Representative flow cytometry images with PI staining for cell apoptosis determination. HepG2 cells were exposed to tamoxifen (20 μM), naringenin (10 and 200 μM) and quercetin (10 and 100 μM) either alone or in combination for 24 h. The cell populations in the sub-G1 phase were calculated to indirectly estimate the cellular apoptotic induction with PI staining using flow cytometry.

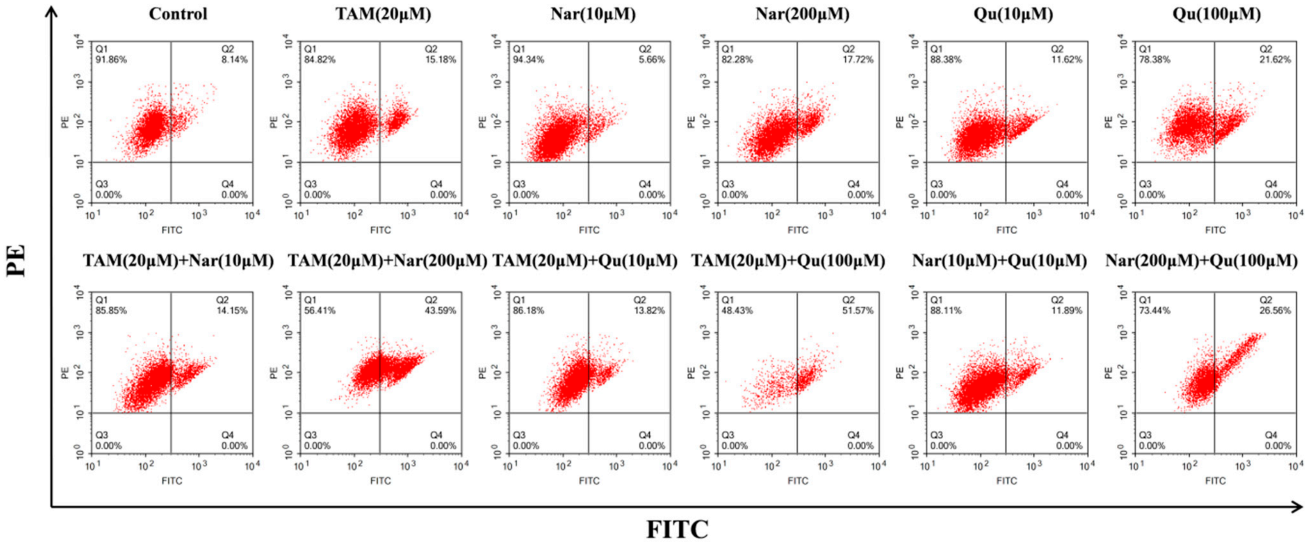

Figure S6. Representative flow cytometry images with JC-1 staining for ΔΨm determination. HepG2 cells were exposed to tamoxifen (20 μM), naringenin (10 and 200 μM) and quercetin (10 and 100 μM) either alone or in combination for 24 h. The loss of ΔΨm was calculated with JC-1 staining using flow cytometry.
